# Fully automated deep learning MAPSE: retrospective analysis and real-time clinical application

**DOI:** 10.1093/ehjimp/qyag087

**Published:** 2026-05-19

**Authors:** Maria Muan Haga, Nora Lindeman Katla, Vegard Holmstrøm, Espen Holte, Stian Stølen, Knut Haakon Stensæth, Andreas Østvik, Lasse Løvstakken, Håvard Dalen, Erik Smistad, Bjørnar Grenne

**Affiliations:** Department of Circulation and Medical Imaging, Norwegian University of Science and Technology, Box 8905 Trondheim 7491, Norway; Department of Circulation and Medical Imaging, Norwegian University of Science and Technology, Box 8905 Trondheim 7491, Norway; Department of Circulation and Medical Imaging, Norwegian University of Science and Technology, Box 8905 Trondheim 7491, Norway; Department of Cardiology and Thoracic Surgery, St.Olavs University Hospital, Trondheim, Norway; Department of Circulation and Medical Imaging, Norwegian University of Science and Technology, Box 8905 Trondheim 7491, Norway; Department of Cardiology and Thoracic Surgery, St.Olavs University Hospital, Trondheim, Norway; Department of Cardiology and Thoracic Surgery, St.Olavs University Hospital, Trondheim, Norway; Department of Circulation and Medical Imaging, Norwegian University of Science and Technology, Box 8905 Trondheim 7491, Norway; Department of Radiology and Nuclear Medicine, St. Olavs University Hospital, Trondheim, Norway; Department of Circulation and Medical Imaging, Norwegian University of Science and Technology, Box 8905 Trondheim 7491, Norway; Department of Cardiology and Thoracic Surgery, St.Olavs University Hospital, Trondheim, Norway; Medical Image Analysis, Health Research, SINTEF Digital, Trondheim, Norway; Department of Circulation and Medical Imaging, Norwegian University of Science and Technology, Box 8905 Trondheim 7491, Norway; Department of Circulation and Medical Imaging, Norwegian University of Science and Technology, Box 8905 Trondheim 7491, Norway; Department of Cardiology and Thoracic Surgery, St.Olavs University Hospital, Trondheim, Norway; Department of Medicine, Levanger Hospital, Nord-Trøndelag Hospital Trust, Levanger, Norway; Department of Circulation and Medical Imaging, Norwegian University of Science and Technology, Box 8905 Trondheim 7491, Norway; Medical Image Analysis, Health Research, SINTEF Digital, Trondheim, Norway; Department of Circulation and Medical Imaging, Norwegian University of Science and Technology, Box 8905 Trondheim 7491, Norway; Department of Cardiology and Thoracic Surgery, St.Olavs University Hospital, Trondheim, Norway

**Keywords:** artificial intelligence, deep learning, echocardiography, MAPSE, real time

## Abstract

**Aims:**

Mitral annular plane systolic excursion (MAPSE) is an accessible echocardiographic measure of left ventricular (LV) function. However, manual measurement methods are operator-dependent and time-consuming. We developed a multistep deep learning (DL) method for off-line and real-time fully automated MAPSE estimation, and aimed to assess agreement, reproducibility, time efficiency, and feasibility compared with standard manual measurements.

**Methods and results:**

The DL-based method was evaluated in two retrospective cohorts (*n* = 1775) and one prospective cohort (*n* = 51). Agreement between DL-MAPSE on B-mode images and experts’ manual M-mode measurements was evaluated in all datasets. Evaluation of test–retest reproducibility, time efficiency using real-time analysis during acquisition, and agreement with cardiac magnetic resonance (CMR)-imaging were performed in subsets of the datasets. DL-MAPSE demonstrated good agreement with manual measurements, with bias 2.9 mm (95% CI 2.8–3.0 mm) and Pearson coefficient 0.81 (95% CI 0.79–0.84) in the primary dataset, and a lower bias of 1.0 mm against CMR-MAPSE compared with −2.1 mm using manual M-mode. Both DL and manual measurements showed good test–retest reproducibility (ICC 0.82 and 0.76, respectively). Real-time DL measurements reduced measurement and acquisition time by 51% (mean 1 min 50 s) per examination. The DL method demonstrated excellent feasibility (96%).

**Conclusion:**

This novel DL method for fully automated MAPSE demonstrated excellent feasibility, robust reproducibility, and good agreement with both manual M-mode and CMR-derived measurements. Automated DL-MAPSE could substantially reduce analysis time and enhance reproducibility, increasing its clinical value as a marker of LV systolic function.

## Graphical Abstract

**Graphical Abstract:**
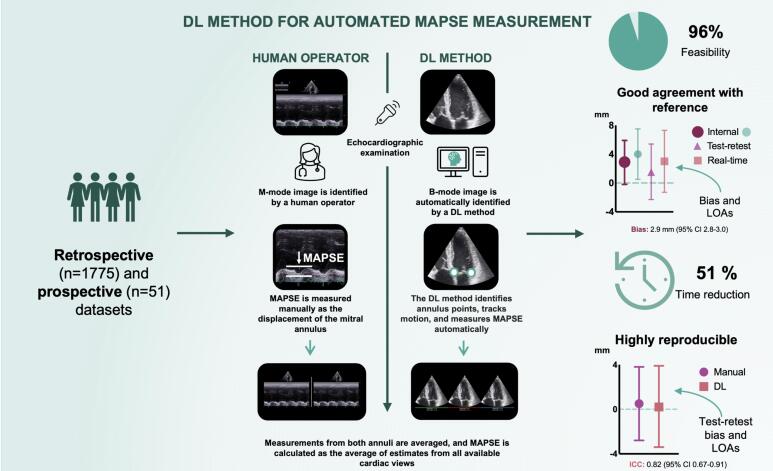
DL: deep learning; MAPSE: mitral annular plane systolic excursion; LOA: limits of agreement; ICC: intraclass correlation.

## Introduction

Echocardiographic assessment of left ventricular (LV) systolic function is essential for diagnosis and management of heart disease. Parameters such as LV ejection fraction (LVEF) and global longitudinal strain (GLS) are commonly used, and recent advances in artificial intelligence are showing promising results in reducing time spent on acquisition and measurement.^1–4^ Nevertheless, both parameters continue to rely on image quality.

Mitral annular plane systolic excursion (MAPSE) measures the displacement of the mitral annular plane during systole. It is a simple marker for LV longitudinal systolic function, and historically recommended as an alternative for LVEF when image quality is poor, as MAPSE only requires visualization of the mitral annulus.^5^ MAPSE is highly reproducible, correlates well with LVEF, and can identify systolic dysfunction in various cardiac diseases.^6–9^ Emerging evidence suggests that MAPSE may also provide prognostic information comparable with GLS and LVEF, and predict adverse outcomes.^10, 11^

MAPSE is measured using motion-mode (M-mode) ultrasound imaging, anatomical M-mode analyses, or two-dimensional speckle tracking echocardiography (2D-STE). However, each method has limitations: m-mode techniques are angle dependent and susceptible to out-of-line motion of the mitral annulus, while 2D-STE MAPSE measurements are time-consuming, require good image quality, and depend on the specific vendor and software utilized.^12^ Given the success of artificial intelligence in automating key echocardiographic parameters, similar approaches may help address these challenges for MAPSE. We have previously developed a deep learning (DL)-based method for automated MAPSE measurement in echocardiography.^13,14^ However, the method has not been clinically validated in large cohorts of patients or for real time use during image scanning.

The aim of this study was to comprehensively assess the agreement, reproducibility, time-efficiency, and feasibility of fully automated DL-based MAPSE compared with manual measurements, evaluating performance in large retrospective datasets, and during real-time acquisition.

## Methods

### Study design

**Figure 1:**
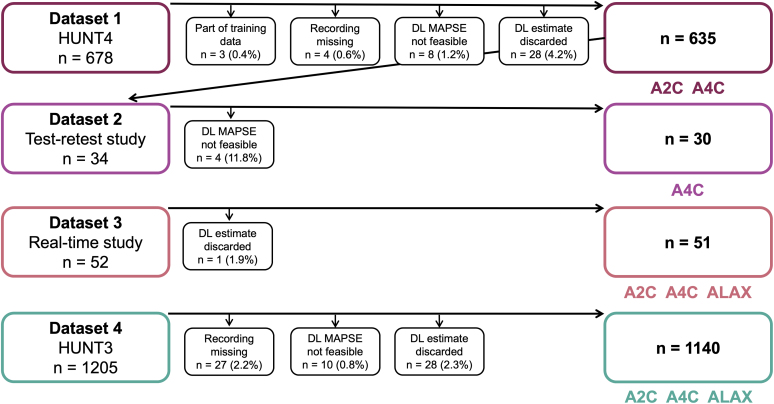
Overview of study design and subject distribution. Study design and distribution of subjects across different datasets. DL, deep learning; MAPSE, mitral annular plane systolic excursion; HUNT, Trøndelag Health Study; A2C, apical two-chamber view; A4C, apical four-chamber view; ALAX, apical long-axis view; *n*, number of subjects.

We performed a method-comparison study, comprising two methods for measuring MAPSE in three separate observational studies with a total of 1856 echocardiographic examinations. We compared the novel DL method against conventional manual reference measurements. First, retrospective images from two population studies were used to evaluate agreement. Then, a test–retest study was performed to evaluate inter-observer reproducibility. Finally, the method’s real-time performance and time-efficiency was evaluated in a prospective cohort (*Figure 1*). Feasibility, as well as correlations between MAPSE, age, and LVEF were examined. Correlation with GLS was evaluated in subsets from the different datasets. To evaluate whether agreement between methods was influenced by LV function, patients were classified into four LV function categories based on LVEF. To assess performance across varying image quality, echocardiographic images in the prospective cohort were graded based on image quality. Validation against an echocardiography-independent modality was performed by assessing agreement with MAPSE derived from cardiac magnetic resonance (CMR) imaging in a subset from the prospective cohort.

### Material

Dataset 1 served as our primary dataset, and included 635 participants randomly selected from the HUNT4 Fitness and Echocardiography study, a substudy from the fourth wave of the Trøndelag Health Survey—HUNT. Inclusion criteria to the substudy were participation in the previous HUNT3 Fitness and Echocardiography substudies or atrial fibrillation, as well as participation in the baseline examinations where all residents ≥ 20 years were invited. Exclusion criteria were severe or recent illness, pregnancy, or restrictions to physical activity ordered by a physician.

Dataset 2 included 30 participants randomly selected from the HUNT4 Fitness and Echocardiography study based on examination date. The participants underwent two separate recording sessions within 15 min of each other. The exams were performed by two different echocardiographers (one sonographer and one cardiologist) blinded to the other.

Dataset 3 included 51 participants recruited from the European Association of Cardiovascular Imaging (EACVI)-accredited Echocardiography Laboratory at St. Olav’s Hospital. The sole exclusion criterion was the need for echocardiographic contrast medium. During these examinations, a real-time application using the DL method enabled live MAPSE measurements during image acquisition. In a randomly selected subgroup of 25 patients, the total time required to obtain MAPSE estimates, including acquisition and analysis time, was evaluated. Image quality was evaluated by the echocardiographic operator during image acquisition using a standardized 4-point scale (0 = excellent, 1 = good, 2 = acceptable, 3 = poor). For 43 patients, CMR imaging was available. CMR-MAPSE could not be measured in one case due to poor image quality, thus images from 42 patients were used to evaluate agreement between CMR-derived MAPSE, DL-MAPSE, and M-mode MAPSE.

Dataset 4 included 1140 participants randomly selected from the HUNT3 Echocardiographic substudy, a substudy from the third wave of the HUNT-study. Exclusion criteria were the use of blood pressure medication, and cardiovascular, pulmonary and malignant disease.^15^ This dataset was included to assess the method’s performance on images acquired with older ultrasound equipment.

All echocardiographic examinations and measurements were performed by experienced personnel in accordance with applicable guidelines.^5^ In Datasets 1 and 2, the echocardiographic images included recordings from the apical 2-chamber view (A2C) and apical 4-chamber view (A4C). In Datasets 3 and 4, the images included recordings from the A2C, A4C, and apical long-axis view (ALAX). For all datasets, focused LV views were used for MAPSE measurements. Echocardiographic images were included regardless of image quality.

LV focused recordings were used for manual measurements of LVEF, LV end-diastolic volume (LVEDV) and LV end-systolic volume (LVESV). The measurements were calculated with Simpson’s biplane disc summation method. GLS was measured using a semiautomatic method based on two-dimensional speckle tracking (2DS, EchoPAC SWO version 204, GE Vingmed Ultrasound, Norway). The mitral inflow peak early to late diastolic velocity ratio (E/A) and the ratio of mitral inflow peak early to mitral annulus early diastolic velocity (E/é) were calculated based on pulsed wave Doppler recordings. All manual analyses were performed blinded to the automated MAPSE measurements. Echocardiographic images were acquired with GE Vivid E95 (Dataset 1–3) and GE Vivid 7 (Dataset 4) scanners (GE Vingmed Ultrasound, Norway). All data were de-identified.

CMR MAPSE was analysed by an expert CMR radiologist, blinded to all other data included echo-MAPSE, using ECG-gated long-axis cine (four-chamber view) as the longitudinal displacement of the septal and lateral mitral annulus between end-diastole (ED) and end-systole (ES). The images were acquired with 1.5 Tesla Magnetom Sola scanner (Siemens Healthineers, Erlangen, Germany). Analysis was conducted using CVI42 software (Circle Cardiovascular Imaging, version 6.0.2).

### MAPSE measurements using the DL method

The in-house DL-based method for fully automated MAPSE measurement consists of four artificial neural networks (see Supplementary data online, *Figure S1*): view classification network, event detection network, landmark detection network, and motion estimation network. The view classification network is trained to recognize different echocardiographic views (including A2C, A4C, and ALAX).^16^ The event detection network identifies image frames corresponding to the diastole and systole, which are used to determine which frames belong to ED and end-systoleES.^17^ Thereafter, the landmark detection network estimates the positions of the mitral annulus in the ED image frame.^14^ Finally, the motion estimation network tracks the mitral annular landmarks from ED to ES using an optical flow-based method.^18^ The network predicts a displacement field between each pair of consecutive images and MAPSE is calculated as the distance between the annular positions in ED and ES. A more detailed technological description of the method has been published previously by our group.^14^ While this technical study used orthogonal projection onto the mitral annular plane normal for MAPSE estimation, this clinical study only uses the distance between the annular positions. Using these networks, a real-time graphical user application was developed and optimized in terms of speed, workflow, and explainability. This application can measure MAPSE retrospectively on ultrasound recordings and prospectively in real-time ultrasound video streaming from a scanner as shown in *Video 1*.

The DL networks have been trained on different datasets. Data from CAMUS, an open French dataset, was used to train the timing network, while the landmark detection network was trained on a subset of data from the HUNT4 study.^19^ The motion estimation network was initially pre-trained on simulated ultrasound data, before being fine-tuned using real patient echocardiographic recordings.^20^

For each patient, the final MAPSE estimate was calculated as the average from up to three heart cycles, from all available cardiac views and walls. In cases where the DL method was not feasible or recordings from all cardiac views were missing, the examination was excluded from the dataset. Feasibility was defined as the DL method’s ability to successfully produce an acceptable estimate of MAPSE for at least one cardiac view. In Datasets 1, 2, and 4, all echocardiographic images were manually reviewed to ensure correct view, timing, and landmark detection, thus ensuring human oversight in accordance with recommendations for trustworthy use of artificial intelligence. In cases where the DL method produced obvious errors, such as landmark detection errors due to incorrect identification of cardiac structures or timing errors, the measurements from the respective cardiac views were discarded and MAPSE was calculated from the remaining available views. Representative examples of discarded predictions are provided in Supplementary data online, *Figure S2*.

### MAPSE measurements using the manual reference method

Manual measurements of MAPSE were performed by expert echocardiographers. For Datasets 1, 2, and 4, reconstructed M-mode images from 2D B-mode recordings (anatomic M-mode) were used. For Dataset 3 (real-time study), MAPSE was measured from M-mode images. MAPSE was measured by aligning the M-mode line through the mitral annulus, and the displacement of each mitral annular point was identified throughout the cardiac cycle. The point of maximal systolic excursion was defined as the peak, whereas ED was defined at mitral valve closure. MAPSE was measured for one heart cycle for each cardiac view, chosen by the operator, and mean MAPSE was calculated by averaging the measurements obtained from all available cardiac walls. All measurements were performed using EchoPAC version 206 (Dataset 1), 204 (Dataset 3), and 113 and 201 (Dataset 4) (GE Vingmed Ultrasound, Norway).

### Ethics

The study was conducted in accordance with the ethical principles of the Declaration of Helsinki and was approved by the Regional Committee for Medical and Health Research Ethics (REC ID 7160, 13083, and 11276), the Data Protection Officer at St. Olavs Hospital, and the Research Council at the Clinic of Cardiology, St. Olavs Hospital. All participants provided written informed consent.

### Statistics

Data are presented as mean ± standard deviation (SD) for normally distributed data, and median with interquartile ranges (IQR) for skewed data. Normality was tested using the Kolmogorov–Smirnov and Shapiro–Wilk tests, along with visual inspection of QQ-plots and histograms. Proportions are presented as numbers (%). To summarize findings across datasets, selected results are reported in ranges. Bland–Altman analyses assessed mean differences (bias) and limits of agreement (LOA). Pearson correlation coefficients were used to evaluate linear correlation. In the test–retest study, reproducibility was evaluated using intraclass correlation coefficients (ICCs) for both methods separately using a linear mixed model. Fisher’s z-transformation tested the significance of correlation differences. A Brown Forsythe test was performed to evaluate difference in variance when measurement pairs were categorized based on LVEF. Data were analysed in IBM SPSS Statistics, v.29 (IBM, New York, NY, USA), StataCorp, v.18.5 (College Station, Texas, USA), and GraphPad Prism v.10.4 (GraphPad Software, Boston, USA). Microsoft’s AI tool Copilot was utilized to assist syntax-coding in statistical analyses, while all code was overseen by the authors.

## Results

Patient characteristics are summarized in *Table 1*. The mean age of participants ranged from 56 to 61 years across the three cohorts, with females comprising 33–52%. The prevalence of cardiac diseases varied between the datasets, with heart failure, atrial fibrillation, and coronary artery disease ranging from 0 to 24%, 0 to 47%, and 0 to 26%, respectively. Participants in Datasets 1, 2, and 4 were predominantly healthy, with a low prevalence of cardiovascular disease, and fewer than 10% had EF below 40%. In contrast, Dataset 3 (real-time study) comprised patients with a broader range of cardiac function.

**Table 1:** Patient characteristics

	Dataset 1 (HUNT4)	Dataset 2 (test–retest)	Dataset 3 (real-time)	Dataset 4 (HUNT3)
**Demographics**				
Participants, *n*	635	30	51	1140
Age, years	61 ± 12	61 ± 13	56 ± 15	49 ± 14
Female, *n*	308 (49%)	12 (40%)	17 (33%)	594 (52%)
**Clinical characteristics**				
Height, cm	172 ± 11	174 ± 9	176 ± 9	172 ± 9
Weight, kg	80 ± 16	84 ± 16	84 ± 17	78.2 ± 14
BMI, kg/m^2^	27 ± 4	28 ± 3	27 ± 5	26 ± 4
BSA, m^2^	1.9 ± 0.2	2.0 ± 0.2	2.0 ± 0.2	1.9 ± 0.2
Heart rate, beats/min	69 ± 12	70 ± 12	71 ± 15	69 ± 11
BP, systolic/diastolic mm Hg	132 ± 18/ 76 ± 10	129 ± 16/ 75 ± 10	133 ± 21/ 81 ± 13	129 ± 16/ 74 ± 11
**Echocardiographic measurements**				
EF, %	57 ± 8	60 ± 7	46 ± 10	58 ± 7
GLS, %			15.9 ± 3.9	16.7 ± 2.4
LVEDV, mL	112 ± 32	119 ± 36	163 ± 45	93 ± 25
LVESV, mL	49 ± 19	48 ± 21	90 ± 39	40 ± 12
E/A	1.0 (0.8–1.3)	1.0 (0.9–1.5)	1.0 ± 0.4	1.4 ± 0.6
E/é	11 ± 3	12 ± 5	9 ± 4	7 ± 2
**LV function by LVEF category**				
Severely reduced: < 30%	1 (0.2%)	0 (0%)	3 (6%)	2 (0.2%)
Moderately reduced: 30–39%	5 (0.8%)	1 (3%)	9 (17%)	12 (1%)
Mildly reduced: 40–49%	54 (9%)	0 (0%)	17 (33%)	114 (10%)
Normal: > 50%	553 (87%)	26 (87%)	22 (42%)	950 (83%)
**Comorbidity**				
Coronary artery disease, *n*	35 (6%)	4 (13%)	13 (26%)	2 (0%)
Heart failure, *n*	12 (2%)	1 (3%)	12 (24%)	0 (0%)
Atrial fibrillation, *n*	127 (20%)	14 (47%)	7 (14%)	0 (0%)
Kidney disease, *n*	20 (3%)	4 (13%)	3 (6%)	19 (2%)
Diabetes, *n*	24 (4%)	5 (17%)	3 (6%)	1 (0%)
Hypertension treatment, *n*	132 (21%)	9 (30%)	19 (37%)	10 (1%)

**Data are presented as mean ± SD, median (IQR) or numbers (%) as relevant.** Twenty-two patients in Dataset 1, 3 patients in Dataset 2, and 62 patients in Dataset 4 had no EF measurements and where thus excluded from LVEF categorization. BMI, body mass index; BSA, body surface area; BP, blood pressure; EF, ejection fraction; GLS, global longitudinal strain; LVEDV, left ventricular end diastolic volume; LVESV, left ventricular end systolic volume; E/A, mitral inflow peak early to late diastolic velocity; E/é, mitral inflow peak early to mitral annulus early diastolic velocity.

In the primary dataset, the mean MAPSE was 11.8 mm (±2.5) when using the DL method, and 14.7 mm (±2.6) with manual measurements. Across the remaining datasets, DL-measured MAPSE ranged from 9.9 to 11.8 mm, while manual values ranged from 12.3 to 15.7 mm. MAPSE measurements by both methods according to datasets and cardiac views are presented in *Table 2*.

**Table 2:** MAPSE measured by the DL method and the manual reference method in the different datasets

	Dataset 1 (HUNT4)			Dataset 2 (Test–retest)			Dataset 4 (Real-time)			Dataset 4 (HUNT3)		
	DL	Manual	Mean diff	DL	Manual	Mean diff	DL	Manual	Mean diff	DL	Manual	Mean diff
Global average	11.8 (2.5)	14.7 (2.6)	2.9*	10.8 (3.2)	12.3 (2.6)	1.3*	9.9 (2.4)	12.9 (2.8)	3.0*	11.6 (1.9)	15.7 (2.4)	4.0*
**LV wall**												
*Septal wall*	11. 5 (2.5)	13.5 (2.8)	2.1*	10.6 (3.2)	11.4 (2.7)	0.8*	9.2 (2.7)	12.0 (2.8)	2.8*	11.9 (1.9)	15.1 (2.9)	3.2*
*Lateral wall*	11.9 (2.9)	15.5 (3.0)	3.6*	11.0 (3.6)	13.2 (2.9)	2.2*	11.0 (2.7)	14.1 (3.2)	3.1*	11.7 (2.3)	16.0 (2.9)	4.3*
*Inferior wall*	12.9 (2.5)	15.3 (2.9)	2.5*				10.0 (2.9)	13.6 (3.7)	3.6*	12.8 (2.0)	16.7 (3.1)	3.9*
*Anterior wall*	11.4 (2.8)	14.7 (3.2)	3.3*				10.2 (2.7)	12.5 (3.2)	2.3*	11.5 (2.5)	15.5 (3.1)	4.0*
*Anteroseptal wall*							8.5 (2.7)	10.8 (3.1)	2.3*	10.5 (2.2)	14.3 (3.1)	3.9*
*Inferolateral wall*							10.3 (2.9)	13.5 (3.1)	3.2*	11.7 (2.3)	16.5 (3.2)	4.9*
**LV views**												
*A4C*	11.7 (2.5)	14.5 (2.6)	2.8*	10.8 (2.8)	12.3 (2.6)	1.3*	10.1 (2.6)	12.7 (2.5)	3.0*	11.8 (1.9)	15.6 (2.6)	3.7*
*A2C*	12.1 (2.5)	15.0 (2.8)	2.9*				10.1 (2.7)	12.8 (3.1)	2.9*	12.1 (2.1)	16.1 (2.8)	4.0*
*ALAX*							9.4 (2.6)	11.9 (2.9)	2.7*	11.1 (2.1)	15.4 (2.8)	4.4*

^*^P < 0.05

Mean (SD) of MAPSE measurements (mm) by both methods, and the mean difference between the two methods grouped by dataset. DL, deep learning; manual, manual method; mean diff, mean difference; A4C, apical four-chamber view; A2C, apical two-chamber view; ALAX, apical long-axis view.

### Agreement between methods

**Figure 2:**
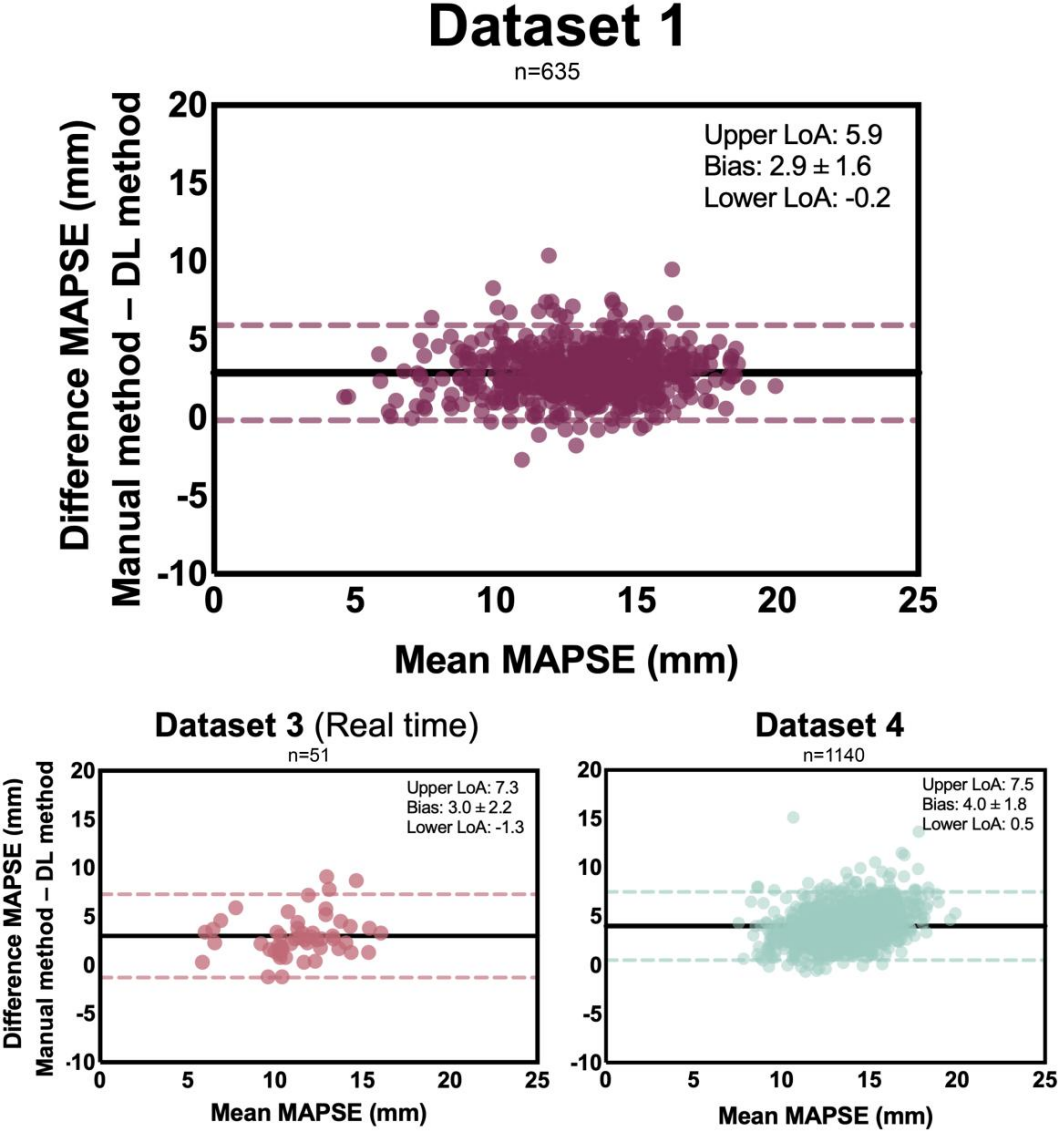
Agreement between manual and DL-based MAPSE across datasets. Bland–Altman plots showing agreement between the DL method and manual measurements in the different datasets. Limits of agreement (LOA) are presented as stapled lines, while the bias is illustrated as a continuous line. MAPSE, mitral annular plane systolic excursion; DL, deep learning; *n*, number of subjects.

**Figure 3:**
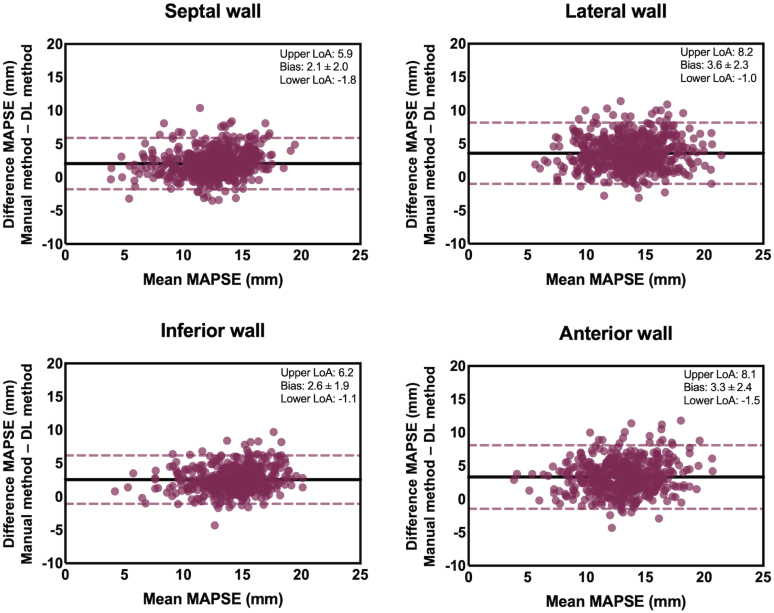
Agreement between manual and DL-based MAPSE by cardiac wall in the primary dataset. Bland–Altman plots for each wall in the primary dataset (Dataset 1, *n* = 635). Limits of agreement (LOA) are presented as stapled lines, while the bias is illustrated as a continuous line. MAPSE, mitral annular plane systolic excursion; DL, deep learning.

The agreement between the DL method and manual measurements in the different datasets is presented in *Figure 2*. In Dataset 1, there was a method-specific bias of 2.9 mm (95% CI 2.8–3.0 mm) with LOA −0.2–5.9 mm. Dataset 3 (real-time study) revealed a similar bias of 3.0 mm (95% CI 2.4–3.6 mm). In Dataset 4, constituting images acquired with a previous-generation scanner, there was a slightly larger bias of 4.0 mm (95% CI 3.9–4.1 mm) and LOA 0.5–7.5 mm. In all datasets, the mean difference across the different cardiac views did not deviate considerably from the average mean difference calculated from all available views (*Figure 3*, Supplementary data online, *Figure S3*). The methods showed a high degree of correlation with manual measurements, with a Pearson coefficient of 0.81 (95% CI 0.79–0.84) in the primary dataset. When running a Brown–Forsythe test, there was no significant difference in variance between measurement pairs labelled by LVEF groups when looking at the agreement between the DL method and the manual method for each patient (*P* = 0.1) (see Supplementary data online, *Figure S4*). Further, no statistically significant differences in agreement were observed between patients with LVEF > 50% and those with LVEF < 50% across all datasets (see Supplementary data online, *Table S1*).

### Reproducibility

**Figure 4:**
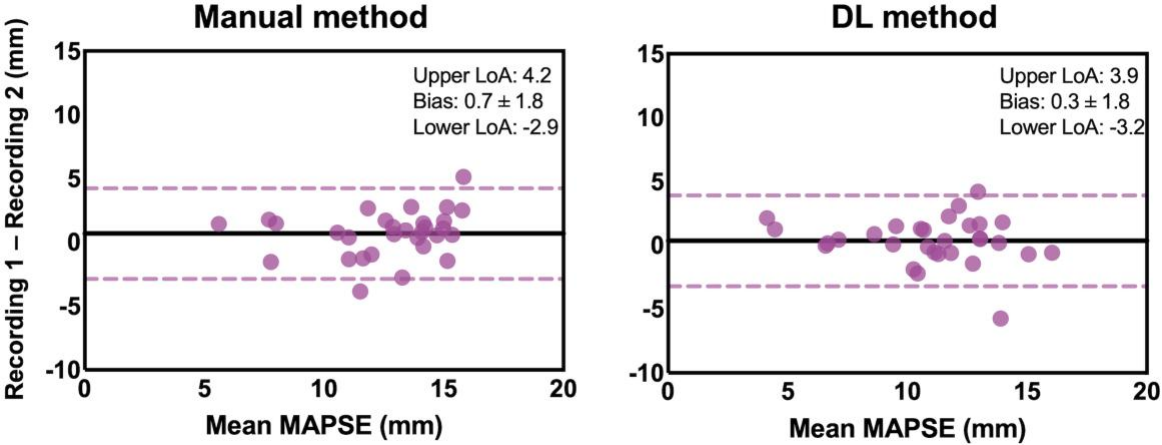
Reproducibility of manual and DL-based MAPSE measurements. Bland–Altman plots showing the mean differences in repeated MAPSE measurements performed on two different recordings using the test–retest dataset (*n* = 30) with the DL method and the manual method. Limits of agreement (LOA) are presented as stapled lines, while the bias is illustrated as a continuous line. MAPSE, mitral annular plane systolic excursion, DL, deep learning.

MAPSE measurements were highly reproducible in the test–retest study, both for the DL and manual method. The repeated DL MAPSE measurements showed a bias of 0.3 (SD ±1.8) mm, whereas the manual method exhibited a bias of 0.7 (SD ±1.8) mm (*Figure 4*). The ICC was 0.82 for the DL method and 0.76 for the manual method, with 95% CI ranging from 0.67 to 0.91 and 0.58 to 0.88, respectively. This difference was not statistically significant (*P* = 0.56).

## Time efficiency

Use of the real-time DL method during image acquisition significantly reduced the time required to obtain MAPSE estimates, with a median time reduction of 1 min 50 s (95% CI 1:40–2:03). The total examination time for all three views with the DL method had a median of 1 min 22 s [IQR 1:09–1:55], compared with 3 min 22 s [IQR 3:01–3:31] for the conventional manual method. Acquisition time with the conventional workflow was 1 min 42 s [IQR 1:27–1:57] and median analysis time was 1 min 37 s [IQR 1:23–1:45].

## Feasibility

**Figure 5:**
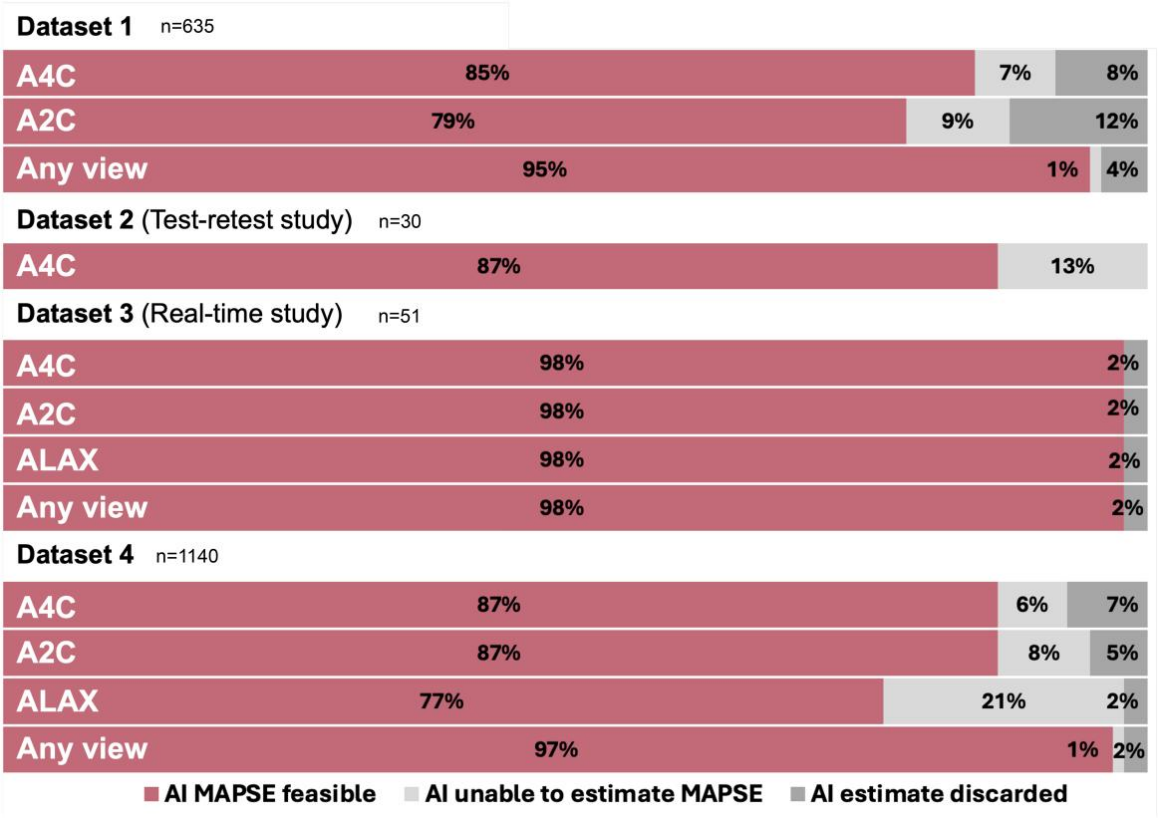
Feasibility of DL-based MAPSE across datasets and cardiac views. Feasibility of the DL method for each cardiac view. ‘DL unable to estimate MAPSE’ means that no MAPSE estimate was produced by the DL method. ‘DL estimate discarded’ means that the measurement was manually discarded during visual quality control due to obvious errors, ensuring human oversight. Patients with missing MAPSE estimates for all views were excluded from the study. DL, deep learning; MAPSE, mitral annular plane systolic excursion; A2C, apical two-chamber view; A4C, apical four-chamber view; ALAX, apical long-axis view; *n*, number of subjects.

The DL method was feasible in 95% (Dataset 1), 88% (Dataset 2: test–retest study), 98% (Dataset 3: real-time study), and 97% (Dataset 4) of the examinations, resulting in an overall feasibility of 96% across all datasets (*Figure 5*). In the cases of non-feasibility, the measurement was either manually discarded during visual quality control due to obvious errors (see Supplementary data online, *Figure S2*), or the DL method failed to produce a MAPSE estimate due to failure to identify ED and ES, or failure to estimate the annular landmark positions with sufficient certainty. Among the 51 patients in the prospective cohort, 16 (32%) had suboptimal image quality, with 9 (18%) rated as acceptable, and 7 (14%) as poor.

## Correlation with GLS, EF, and age

Both DL-measured and manually measured MAPSE showed a moderate to strong correlation with GLS, while there was a weak to moderate correlation with EF. Further, both methods showed a weak to moderate negative correlation with age in all datasets. Detailed results are presented in Supplementary data online, *Table S2*.

## Comparison with CMR

Comparison with CMR-derived MAPSE showed a bias of 1.0 mm (95% CI 0.1–1.9, LOA −4.5–6.5 mm) for the DL method compared with a bias of −2.1 mm (95% CI −2.8–−1.4, LOA −6.6–2.4 mm) for the manual method (see Supplementary data online, *F*igure *S5*). Both DL-MAPSE and M-mode MAPSE showed moderate correlation with CMR MAPSE, with no significant difference between the methods (*P* = 0.15, Pearson correlation coefficients 0.5 and 0.7, respectively).

## Discussion

This study demonstrates the clinical feasibility of a multi-step DL method for fully automated MAPSE estimation directly from B-mode images, with the aim of assessing its agreement, reproducibility, efficiency, and feasibility compared with manual M-mode measurements. To our knowledge, this is the first study that examines the use of real-time automatic MAPSE during transthoracic echocardiography and the largest validation study of DL-based MAPSE measurements. Across diverse cohorts, the DL method showed good agreement with manual measurements, with a small bias likely related to methodological differences. The method was highly reproducible, reduced the time to obtain MAPSE by 51% compared with the conventional manual workflow and proved feasible in 96% of examinations.

### Agreement

Biases between the DL-based and manual MAPSE measurements ranged from 2.9 to 4.0 mm in the different datasets, indicating a systematic lower estimation of MAPSE with the DL method. This difference is likely predominantly a result of inherent differences between the two methods: with conventional manual measurements, MAPSE is measured along the M-mode line, extending from the transducer through the mitral annulus, guided by 2D B-mode images. Because the annulus usually has an inward/translational motion during systole, the measured distance along the M-mode line often represents both the true longitudinal annulus displacement and a translational motion, resulting in overestimation of MAPSE. Additionally, as M-mode measurements are angle dependent, a non-aligned M-mode line may add to overestimation of the true MAPSE due to angular deviation from the direction of motion of the mitral annulus.^14^ In contrast, the DL method measures MAPSE directly from B-mode images, tracking the mitral annular hinge points from ES to ED, thus representing the actual displacement of the annulus (see Supplementary data online, *Figure S6*). Furthermore, when compared with CMR-derived MAPSE, the echocardiography-derived DL method demonstrated a smaller bias than the manual M-mode method, although LOAs remained wide and correlations were modest for both approaches. The lower bias indicates that the DL method, which tracks the anatomical annulus, provides more correct MAPSE representation than M-mode, with its inherent angle-dependency and challenges with translational motion. Lastly, in addition to the methodological differences, potential limitations with the DL method, such as timing- and tracking-related inaccuracies, cannot be excluded.

Agreement between methods was generally consistent across all datasets, but a greater bias was observed in Dataset 4. Different factors could contribute, such as differences in temporal resolution between M-mode and B-mode, and greater susceptibility of conventional M-mode to misalignment compared with reconstructed M-mode from B-mode images. These factors may explain the greater bias seen in Dataset 4, which used traditional M-mode as reference instead of the reconstructed M-mode images from 2D B-mode used for the other datasets.

There was a high correlation between DL-based and manual measurements, and a low standard deviation (1.9 mm) of the bias across all datasets, indicating that our method produces precise and consistent MAPSE estimates. For comparison, previously published studies aiming to establish reference values for MAPSE have reported standard deviations around 3.0 mm for manual MAPSE in measurements across populations, suggesting that some degree of variation is expected in clinical practice.^12^

There are, to our knowledge, only a few studies assessing automated methods for measuring MAPSE in transthoracic echocardiography. Storve *et al*. proposed an automatic algorithm for measuring MAPSE using an automatic segmentation method combined with tissue Doppler mode to track the mitral annular points, which resulted in a bias of −0.6 (SD ±2.1) mm.^21^ A formerly described DL-based method by Smistad *et al*. showed a resembling bias of −0.9 (SD ±4.6) mm.^13^ Compared with these previous methods, our method exhibits a larger bias, which is likely explained by fundamental methodological differences as discussed. Furthermore, our approach offers several advantages, such as fully automated measurement from B-mode images for three different cardiac views, without requiring Doppler and ECG, as well as real-time application.

### Reproducibility

Our study shows excellent inter-observer reproducibility for MAPSE, both measured automatically and manually, comparable with previously reported results.^12,22^ In contrast to the majority of earlier studies that examined repeated analyses on the same images, our test–retest dataset, using echocardiograms acquired by different operators, better captures the variability encountered in clinical practice. Both methods showed low variability and a high degree of consistency, with the DL method achieving a numerically lower bias and a slightly higher ICC. Given that repeated measurements on the exact same image by a DL method always produce the same result, the variability observed is likely due to a combination of the image acquisition process, the DL algorithm, and physiological variation. The slightly better reproducibility with the DL method may be attributed to its use of multi-cycle averaging, as opposed to the single heart cycle used for conventional measurements.

### Time efficiency

The results of this study demonstrate the potential of the DL-based method to perform fully automated measurements in real-time during echocardiographic examinations.^23^ Consequently, we observed a 51% reduction in the time used to acquire a MAPSE estimate with the DL method compared with conventional acquisitions plus offline manual measurements. The DL method’s efficiency allows effortless averaging of measurements across multiple heart cycles, which may be particularly beneficial for patients with atrial fibrillation and other arrhythmias. Real-time measurements improve echocardiographic workflow by enabling immediate assessment during image acquisition and eliminating the need for time-consuming manual analyses.

### Feasibility

The proposed DL method in this study showed an overall feasibility of 96%. When comparing the feasibility between datasets, the highest feasibility was found in the real-time dataset (Dataset 3), with feasibility of 98% achieved across all three cardiac views. This illustrates the potential advantage of a DL-based method that provides visual feedback to the operator during image acquisition, allowing optimization before storing images. This transparent, multi-step approach enables the operator to review, challenge, and ultimately trust the DL MAPSE measurements. Therefore, the high feasibility seen in Dataset 3 might be most representative to reflect what is achievable in clinical practice. Additionally, 32% in the prospective cohort had suboptimal image quality. Despite this, DL-MAPSE showed high feasibility and maintained agreement comparable with other cohorts, indicating that DL-MAPSE is both feasible and robust under more challenging clinical circumstances. Exempting Dataset 3, though overall feasibility was high, the feasibility for each view was lower. During manual quality control of images and measurements, a frequent reason for non-feasibility was the image being out of sector, thus displaying more of, or other structures than those normally seen in the standard LV-focused views. This can be explained by such images being underrepresented in the training data of the DL model.^14^

### Correlation with GLS, EF, and age

We found a moderate to strong correlation between MAPSE and GLS, consistent with previous studies, although there has been considerable variability.^24–27^ Interestingly, in the real-time study, automatically measured MAPSE showed a higher correlation with GLS than manual measurements, a finding that may indicate a more sensitive reflection of LV function, though this should be interpreted with caution given the small sample size. Previous studies have found stronger correlations of GLS with B-mode and 2D-STE MAPSE than with M-mode, though none as high as observed in this study, in which DL measurements were derived using an optical flow-based network for motion estimation.^12,22^ Both DL and manual MAPSE showed a similar negative correlation with age, aligning with prior findings that MAPSE declines with age, thus indicating that both methods similarly reflect age-related decline in longitudinal function.^28,29^ Lastly, the predominantly healthy study population may have contributed to the weak correlation observed between MAPSE and EF in this study.

### Clinical implications

MAPSE is a simple and reliable parameter of LV function that has been claimed to be underused in clinical practice due to limitations of the M-mode technique.^30^ This study highlights the potential of a DL-based approach to automate and expedite the echocardiographic examination, while reducing variability and operator dependency. Importantly, as the operator is able to evaluate the performance during real-time acquisition, the method effectively enhances confidence in the measurements. Our findings show that fully automated MAPSE measurement in real time is both feasible, accurate, and reproducible, with the potential to enhance clinical workflow and reduce examination time. Future implementation into clinical practice could enhance the value of MAPSE as a parameter for evaluation of LV systolic function, particularly in patients with challenging image quality. Furthermore, the ability to automate analyses of images in large existing databases could have considerable implications for future research.

### Strengths and limitations

A key limitation is the absence of an established gold standard for MAPSE, making it difficult to define which method is more accurate. Comparison with CMR revealed slightly better agreement with DL-MAPSE than with M-mode MAPSE, but the small sample size limits the strength of the results, and the findings do not solve the variation introduced by methodological differences.

As for all DL-based methods, another limitation is the reliance on training data. While all examinations used for training of the model were excluded, the use of data from HUNT3 and HUNT4 may have introduced demographic similarities. However, comparable results from Dataset 3 (real-time study), which was not used for training and comprised other patient categories, supports the method’s generalizability. Furthermore, although different generations of ultrasound scanners were used, all images were acquired using machines from one single vendor. Another limitation is that measurements from three different cardiac views were available only in Datasets 3 and 4, whereas the remaining datasets contained measurements from either one or two views. Further, measurements were performed within the same geographic region, and most participants in Datasets 1, 2, and 4 had preserved LV function and a low prevalence of cardiac disease. This limits the generalizability of the findings, particularly for populations with cardiac dysfunction or heart failure. Future studies should include external validation in an independent database and evaluation in more diverse population groups that includes a broader range of demographic characteristics and cardiac function. However, Dataset 3 comprised a prospectively recruited, unselected clinical cohort encompassing a wide range of image quality and more diverse cardiac function, thereby strengthening the real-world applicability of the results.

Other strengths include the use of separate datasets with different generations of ultrasound machines and different operators, with consistent results across the cohorts. Large populations with an equal distribution of sexes further enhance robustness.

## Conclusions

A novel DL method for measuring MAPSE directly from B-mode images was feasible, time efficient, and had good agreement with both manual M-mode and CMR-derived measurements. Further, the method provided highly reproducible measurements and can therefore reduce the variability seen in manual measurements. The DL application can be utilized in real-time during echocardiographic scanning, potentially improving the clinical workflow. These results suggest that implementation of the DL-based method could strengthen the clinical value of MAPSE as an easily available LV parameter.

## Supplementary Material

qyag087_Supplementary_Data

## Data Availability

The dataset can be made available from the corresponding author upon reasonable request.

## Lead authors biography

Maria Muan Haga and Nora Lindeman Katla are final-year medical students at the Norwegian University of Science and Technology (NTNU). As part of their student thesis, they became part of a research group at the Department of Circulation and Medical Imaging, NTNU. Their research focuses on application of artificial intelligence in echocardiography.

## References

[1] Tromp J, Seekings PJ, Hung C-L, Iversen MB, Frost MJ, Ouwerkerk W et al Automated interpretation of systolic and diastolic function on the echocardiogram: a multicohort study. Lancet Digit Health 2022;4:e46–54. 34863649 10.1016/S2589-7500(21)00235-1

[2] Salte I, Østvik A, Smistad E, Melichova D, Nguyen T, Karlsen S et al Artificial intelligence for automatic measurement of left ventricular strain in echocardiography. JACC Cardiovasc Imaging 2021;14:1918–28. 34147442 10.1016/j.jcmg.2021.04.018

[3] Ouyang D, He B, Ghorbani A, Yuan N, Ebinger J, Langlotz CP et al Video-based AI for beat-to-beat assessment of cardiac function. Nature 2020;580:252–6. 32269341 10.1038/s41586-020-2145-8 PMC8979576

[4] Holmstrøm V, Smistad E, Stølen S, Holte E, Løvstakken L, Dalen H et al Real-time global longitudinal strain during echocardiography: a deep learning platform for improved workflow. J Am Soc Echocardiogr 2025;38:1041–51. 40876495 10.1016/j.echo.2025.08.015

[5] Lang RM, Badano LP, Mor-Avi V, Afilalo J, Armstrong A, Ernande L et al Recommendations for cardiac chamber quantification by echocardiography in adults: an update from the American society of echocardiography and the European association of cardiovascular imaging. Eur Heart J Cardiovasc Imaging 2015;16:233–71. 25712077 10.1093/ehjci/jev014

[6] Matos J, Kronzon I, Panagopoulos G, Perk G. Mitral annular plane systolic excursion as a surrogate for left ventricular ejection fraction. J Am Soc Echocardiogr 2012;25:969–74. 22795199 10.1016/j.echo.2012.06.011

[7] Xiao HB, Kaleem S, McCarthy C, Rosen SD. Abnormal regional left ventricular mechanics in treated hypertensive patients with ‘normal left ventricular function’. Int J Cardiol 2006;112:316–21. 16309760 10.1016/j.ijcard.2005.10.001

[8] Alam M. The atrioventricular plane displacement as a means of evaluating left ventricular systolic function in acute myocardial infarction. Clin Cardiol 1991;14:588–94. 1747969 10.1002/clc.4960140711

[9] Alam M, Höglund C, Thorstrand C, Philip A. Atrioventricular plane displacement in severe congestive heart failure following dilated cardiomyopathy or myocardial infarction. J Intern Med 1990;228:569–75. 2280234 10.1111/j.1365-2796.1990.tb00281.x

[10] Xue H, Artico J, Davies RH, Adam R, Shetye A, Augusto JB et al Automated in-line artificial intelligence measured global longitudinal shortening and mitral annular plane systolic excursion: reproducibility and prognostic significance. J Am Heart Assoc 2022;11:e023849. 35132872 10.1161/JAHA.121.023849 PMC9245823

[11] Falconer D, Fröjdh F, Brieger D, Captur G, Kozor R, Ugander M. The diagnostic and prognostic utility of mitral annular plane systolic excursion (MAPSE)–a systematic review and meta-analysis. medRxiv 25323051, 10.1101/2025.03.05.25323051, 5 March 2025, preprint: not peer reviewed. PMC13477369 42396784

[12] Wang Y-H, Sun L, Li S-W, Wang C-F, Pan X-F, Liu Y et al Normal reference values for mitral annular plane systolic excursion by motion-mode and speckle tracking echocardiography: a prospective, multicentre, population-based study. Eur Heart J Cardiovasc Imaging 2023;24:1384–93. 37530466 10.1093/ehjci/jead187 PMC10531139

[13] Smistad E, Østvik A, Salte IM, Leclerc S, Bernard O, Lovstakken L, eds. Fully automatic real-time ejection fraction and MAPSE measurements in 2D echocardiography using deep neural networks. In: 2018 IEEE International Ultrasonics Symposium (IUS). Kobe, Japan: IEEE; 2018. p1–4.

[14] Smistad E, Østvik A, Grue JF, Dalen H, Lovstakken L, eds. Tracking-based mitral annular plane systolic excursion (MAPSE) measurement using deep learning in B-mode ultrasound. In: 2022 IEEE International Ultrasonics Symposium (IUS). Venice, Italy: IEEE; 2022. p1–4.

[15] Letnes JM, Eriksen-Volnes T, Nes B, Wisløff U, Salvesen Ø, Dalen H. Variability of echocardiographic measures of left ventricular diastolic function. The HUNT study. Echocardiography 2021;38:901–8. 33960012 10.1111/echo.15073

[16] Østvik A, Smistad E, Aase SA, Haugen BO, Lovstakken L. Real-time standard view classification in transthoracic echocardiography using convolutional neural networks. Ultrasound Med Biol 2019;45:374–84. 30470606 10.1016/j.ultrasmedbio.2018.07.024

[17] Fiorito AM, Østvik A, Smistad E, Leclerc S, Bernard O, Lovstakken L, eds. Detection of cardiac events in echocardiography using 3D convolutional recurrent neural networks. In: 2018 IEEE International Ultrasonics Symposium (IUS). Kobe, Japan: IEEE; 2018. p1–4.

[18] Østvik A, Salte IM, Smistad E, Nguyen TM, Melichova D, Brunvand H et al Myocardial function imaging in echocardiography using deep learning. IEEE Trans Med Imaging 2021;40:1340–51. 33493114 10.1109/TMI.2021.3054566

[19] Leclerc S, Smistad E, Pedrosa J, Østvik A, Cervenansky F, Espinosa F et al Deep learning for segmentation using an open large-scale dataset in 2D echocardiography. IEEE Trans Med Imaging 2019;38:2198–210. 30802851 10.1109/TMI.2019.2900516

[20] Salte IM, Østvik A, Olaisen SH, Karlsen S, Dahlslett T, Smistad E et al Deep learning for improved precision and reproducibility of left ventricular strain in echocardiography: a test-retest study. J Am Soc Echocardiogr 2023;36:788–99. 36933849 10.1016/j.echo.2023.02.017

[21] Storve S, Grue JF, Samstad S, Dalen H, Haugen BO, Torp H. Realtime automatic assessment of cardiac function in echocardiography. IEEE Trans Ultrason Ferroelectr Freq Control 2016;63:358–68. 26780792 10.1109/TUFFC.2016.2518306

[22] Hensel KO, Roskopf M, Wilke L, Heusch A. Intraobserver and interobserver reproducibility of M-mode and B-mode acquired mitral annular plane systolic excursion (MAPSE) and its dependency on echocardiographic image quality in children. PLoS One 2018;13:e0196614. 29746603 10.1371/journal.pone.0196614 PMC5944962

[23] Smistad E, Østvik A, Pedersen A. High performance neural network inference, streaming, and visualization of medical images using FAST. IEEE access 2019;7:136310–21.

[24] Støylen A, Mølmen HE, Dalen H. Relation between mitral annular plane systolic excursion and global longitudinal strain in normal subjects: the HUNT study. Echocardiography 2018;35:603–10. 29399886 10.1111/echo.13825

[25] Wenzelburger FW, Tan YT, Choudhary FJ, Lee ES, Leyva F, Sanderson JE. Mitral annular plane systolic excursion on exercise: a simple diagnostic tool for heart failure with preserved ejection fraction. Eur J Heart Fail 2011;13:953–60. 21807660 10.1093/eurjhf/hfr081

[26] Chiu DY, Abidin N, Hughes J, Sinha S, Kalra PA, Green D. Speckle tracking determination of mitral tissue annular displacement: comparison with strain and ejection fraction, and association with outcomes in haemodialysis patients. Int J Cardiovasc Imaging 2016;32:1511–8. 27464963 10.1007/s10554-016-0946-5

[27] Smiseth OA, Rider O, Cvijic M, Valkovič L, Remme EW, Voigt J-U. Myocardial strain imaging: theory, current practice, and the future. Cardiovascular Imaging 2025;18:340–81. 39269417 10.1016/j.jcmg.2024.07.011

[28] Wandt B, Bojö L, Hatle L, Wranne B. Left ventricular contraction pattern changes with age in normal adults. J Am Soc Echocardiogr 1998;11:857–63. 9758377 10.1016/s0894-7317(98)70005-7

[29] Støylen A, Dalen H, Molmen HE. Left ventricular longitudinal shortening: relation to stroke volume and ejection fraction in ageing, blood pressure, body size and gender in the HUNT3 study. Open Heart 2020;7:e001243. 32978265 10.1136/openhrt-2020-001243 PMC7520903

[30] Cirin L, Crișan S, Luca C-T, Buzaș R, Lighezan DF, Văcărescu C et al Mitral annular plane systolic excursion (MAPSE): a review of a simple and forgotten parameter for assessing left ventricle function. J Clin Med 2024;13:5265. 39274478 10.3390/jcm13175265 PMC11396218

