# Supplementary material for: Fully automated deep learning MAPSE: retrospective analysis and real-time clinical application
Source: Eur Heart J Imaging Methods Pract. 2026 May 19;4(1):qyag087. doi: 10.1093/ehjimp/qyag087 (PMC13403282; doi:10.1093/ehjimp/qyag087)

# Supplementary materials

## Supplementary tables

**Supplementary table 1:** **MAPSE measured by the DL method and the manual reference method across the different datasets, categorized by LVEF**

|  | **Dataset 1** | | | **Dataset 3** | | | **Dataset 4** | | |
| --- | --- | --- | --- | --- | --- | --- | --- | --- | --- |
|  | **DL** | **Manual** | **Mean diff** | **DL** | **Manual** | **Mean diff** | **DL** | **Manual** | **Mean diff** |
| MAPSE  (EF  < 50%) | 9.4 (±2.8) | 12.4 (±3.1) | 3.0* (±1.4) | 9.0 (±2.1) | 11.9  (±2.6) | 2.9*  (±1.8) | 10.6 (±1.9) | 14.7 (±2.5) | 4.1*  (±1.8) |
| MAPSE  (EF  *≥* 50%) | 12.1 (±2.1) | 14.9 (±2.4) | 2.8* (±1.5) | 11.0 (±2.4) | 14.1 (±2.7) | 3.1*  (±2.7) | 11.8 (±1.8) | 15.8 (±2.4) | 4.0*  (±1.8) |

*Mean (SD) of MAPSE measurements (mm) obtained by both methods, and the mean difference between the two methods, grouped by ejection fraction (EF ≥ 50% and EF < 50%).
DL: deep learning; Manual: manual method; Mean diff: mean difference.
*The difference in agreement (mean difference) between patients with EF < 50% and those with EF ≥* 50% *was not statistically significant. The corresponding p-values were 0.5 (Dataset 1), 0.5 (Dataset 3), and 0.4 (Dataset 4).*

**Supplementary table 2: Correlation analyses with EF, GLS, and age**

|  | **MAPSE** | **Correlation with EF** | **Correlation with GLS** | **Correlation with age** |
| --- | --- | --- | --- | --- |
| **Dataset 1** (HUNT4) | **DL** | 0.31 (0.24 – 0.38) |  | -0.40  (-0.46 – -0.33) |
|  | **Manual** | 0.30 (0.23 – 0.37) |  | -0.39  (-0.46 – -0.33) |
|  | **P-value difference** | P = 0.85 |  | P = 0.83 |
| **Dataset 3** (Real-time study) | **DL** | 0.59 (0.38 – 0.74) | 0.74 (0.58 – 0.84) | Not significant |
|  | **Manual** | 0.51 (0.27 – 0.69) | 0.52 (0.29 – 0.70) | Not significant |
|  | **P-value difference** | P = 0.57 | P = 0.07 | P = 0.96 |
| **Dataset 4** (HUNT3) | **DL** | 0.27 (0.21 – 0.32) | 0.38 (0.33 – 0.43) | - 0.36  (-0.41 – -0.31) |
|  | **Manual** | 0.17 (0.11 – 0.23) | 0.41 (0.36 – 0.46) | - 0.52  (-0.56 – -0.48) |
|  | **P-value difference** | P = 0.01 | P = 0.40 | P < 0.01 |
| **Global** | **DL** | 0.32 (0.28 – 0.37) | 0.42 (0.36 – 0.46) | -0.31 (-0.35 – -0.27) |
|  | **Manual** | 0.25 (0.21 – 0.29) | 0.42 (0.37 – 0.46) | -0.49  (-0.62 – -0.32) |
|  | **P-value difference** | P = 0.02 | P = 1 | P <0.01 |
| *Correlation analyses between MAPSE and EF, GLS and age. Presented as Pearson correlation coefficients (95% CI intervals). P-value difference shows the significance of difference in correlation between the DL method and manual method.  DL= deep learning, EF = ejection fraction, GLS = global longitudinal strain.* | | | | |

## Supplementary figure legends

**Supplementary figure 1:** Illustration of the four artificial neural networks that make up the DL method used for automatically measuring MAPSE. ES: end-systole; ED: end-diastole; MAPSE: mitral annular plane systolic excursion.

**Supplementary figure 2:** Representative images discarded during visual quality control due to errors. The two green “dots” represent the DL model’s attempt to detect the positions of the mitral annuli. In picture A, the DL method identified the mitral annuli in the completely wrong locations. In picture B, the lateral annulus is correctly identified, while the method was not able to detect the septal annulus. In picture C, the DL method identified only one of the two points. In picture D, the echocardiographic image is partly out of sector, thus the DL model does not have the opportunity to find the posterior-inferior annulus.

**Supplementary figure 3:** Bland-Altman plots for each wall in the secondary datasets (Dataset 3 and 4). Limits of agreement (LOA) are presented as stapled lines, while the bias is illustrated as a continuous line. MAPSE: mitral annular plane systolic excursion; DL: deep learning.

**Supplementary figure 4:** Bland-Altman plot illustrating measurement pairs labeled by left ventricular ejection fraction. Limits of agreement (LOA) are presented as stapled lines, while the bias is illustrated as a continuous line. MAPSE: mitral annular plane systolic excursion; DL: deep learning.

**Supplementary figure 5:** Bland-Altman plots showing agreement between CMR-derived MAPSE, DL-MAPSE and manual M-mode MAPSE. Limits of agreement (LOA) are presented as stapled lines, while the bias is illustrated as a continuous line. MAPSE: mitral annular plane systolic excursion; DL: deep learning; A4C: apical four-chamber view; MRI: magnetic resonance imaging; CMR: cardiac magnetic resonance.

**Supplementary figure 6:** An illustration of important differences in measurement techniques. The M-mode technique captures the movement along the M-mode line. During cardiac contraction, the mitral annuli move not only toward the apex in a linear direction but also inwards. This latter movement does not align with the M-mode line placed in end-diastole, potentially resulting in translation of the annulus and consequently overestimation of longitudinal deformation. In contrast, the DL method tracks the movement of the mitral annuli throughout the cardiac cycle, providing a measurement of the actual movement of the mitral annuli.

## Supplementary figure 1

##
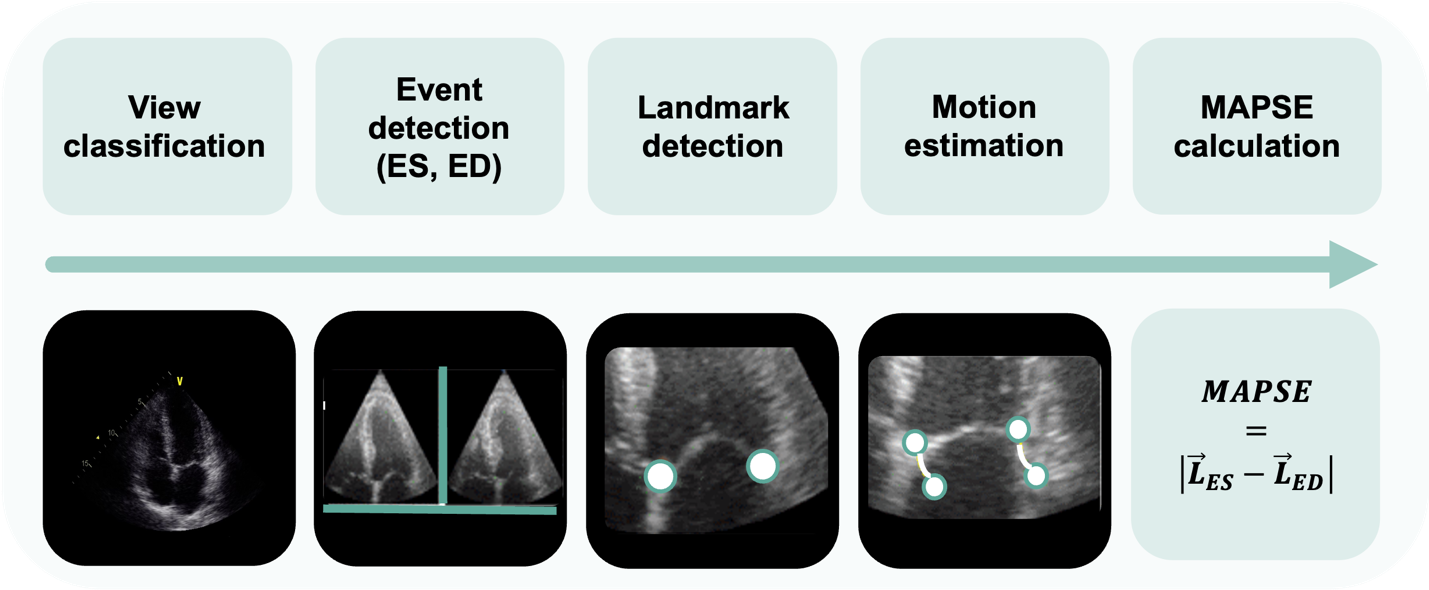


## Supplementary figure 2


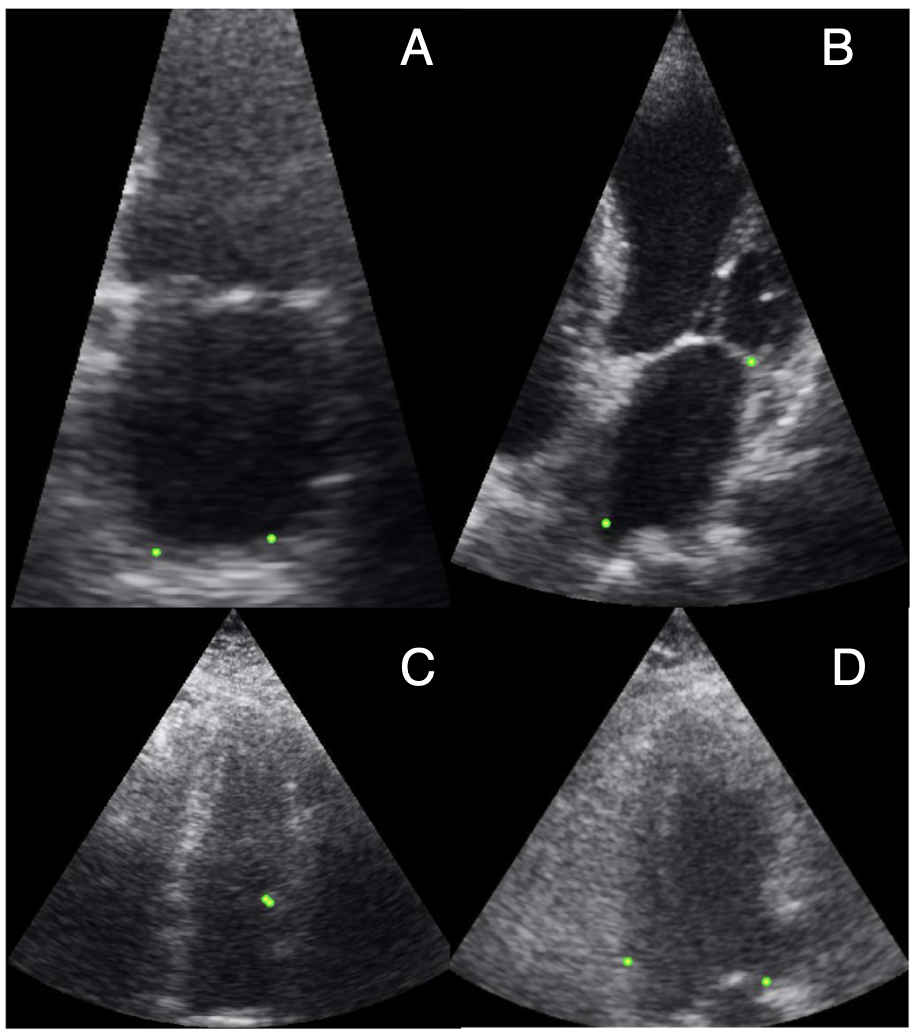


## Supplementary figure 3


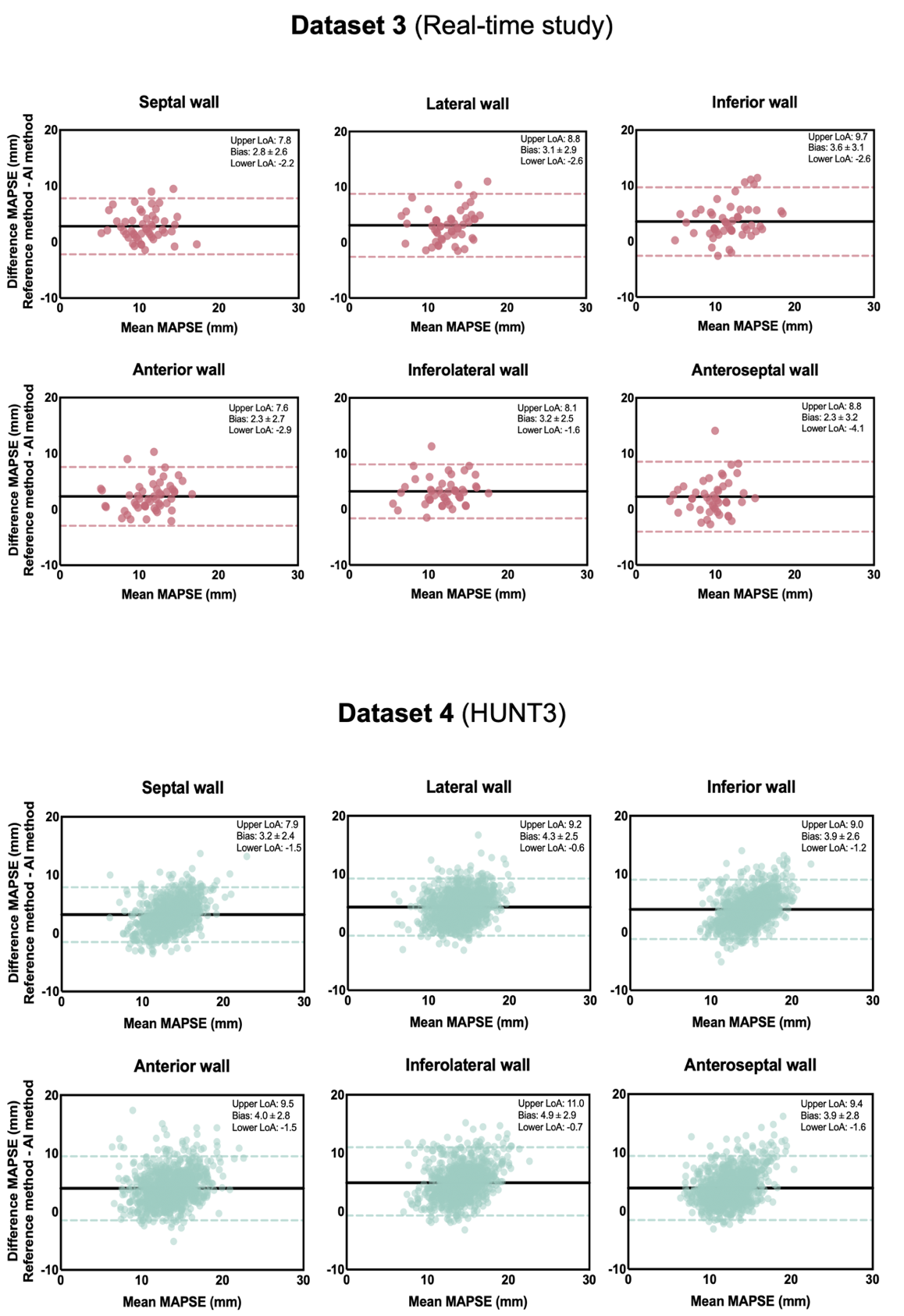


## Supplementary figure 4


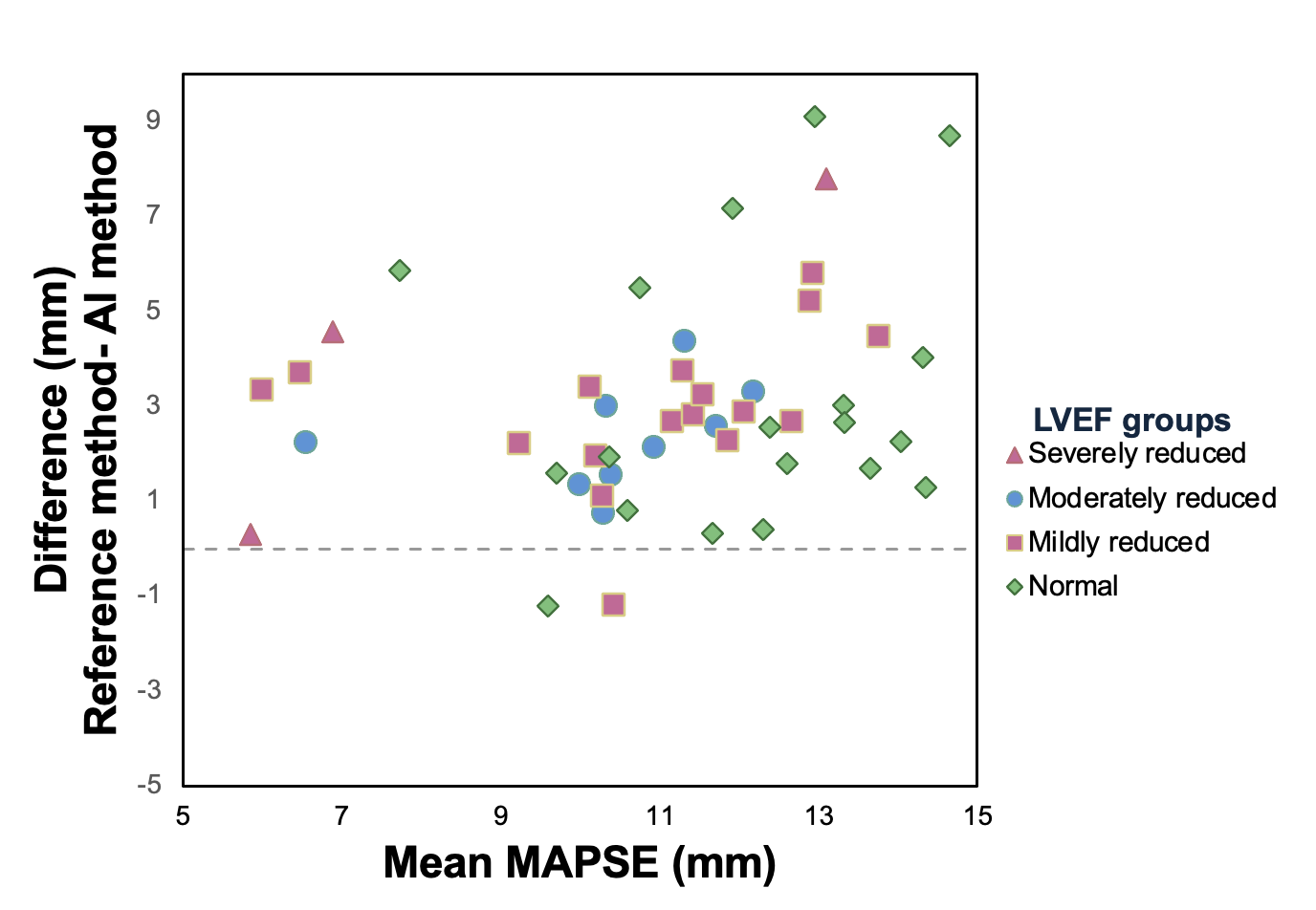


## Supplementary figure 5


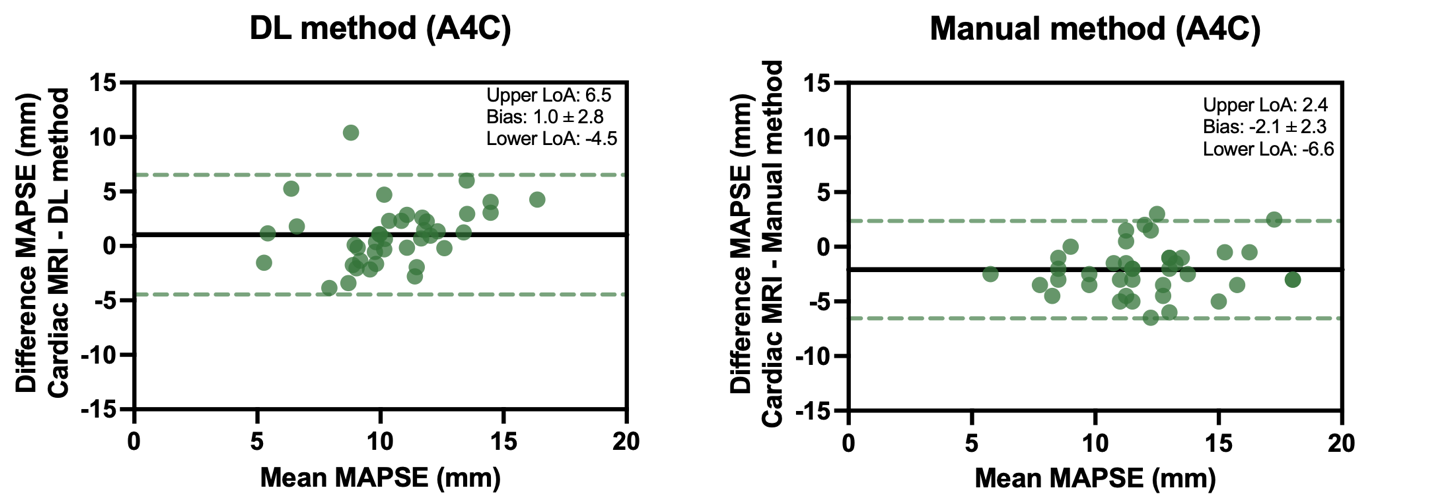


## Supplementary figure 6


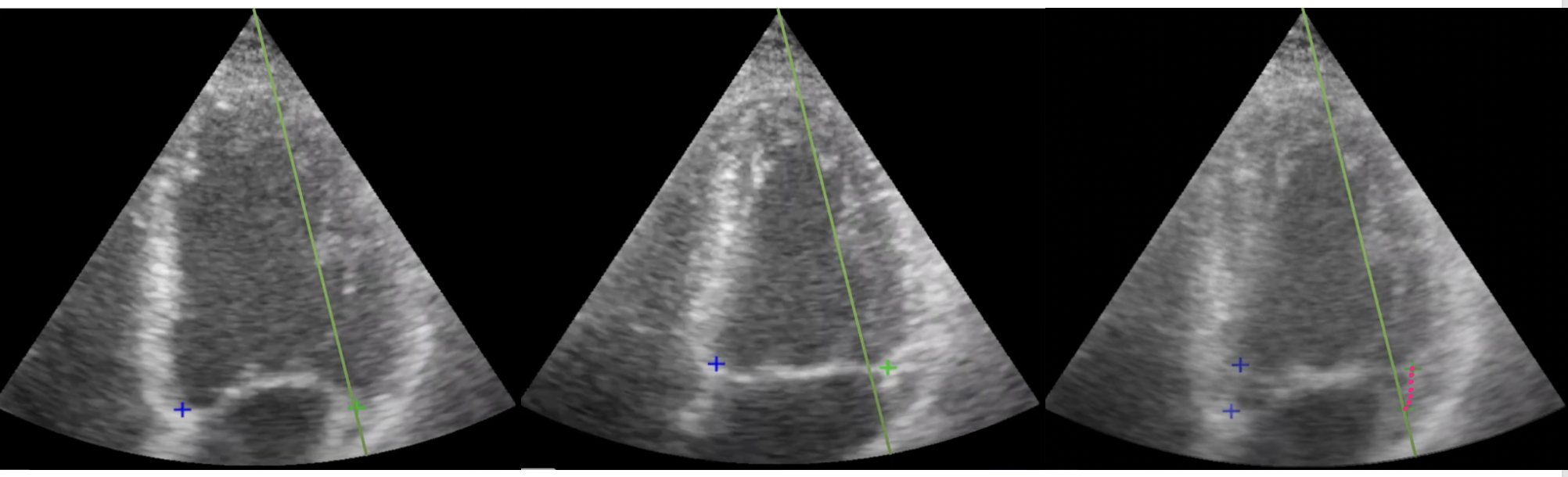

Supplement: qyag087_Supplementary_Data [file qyag087_Supplementary_Data.docx]
